# Identification and functional characterization of copy number variations in diverse chicken breeds

**DOI:** 10.1186/1471-2164-15-934

**Published:** 2014-10-25

**Authors:** Ruili Han, Pengkun Yang, Yadong Tian, Dandan Wang, Zengxuan Zhang, Lele Wang, Zhuanjian Li, Ruirui Jiang, Xiangtao Kang

**Affiliations:** College of Animal Science and Veterinary Medicine, Henan Agricultural University, Henan Innovative Engineering Research Center of Poultry Germplasm Resources, Zhengzhou, Henan 450002 China

## Abstract

**Background:**

The detection and functional characterization of genomic structural variations are important for understanding the landscape of genetic variation in the chicken. A recently recognized aspect of genomic structural variation, called copy number variation (CNV), is gaining interest in chicken genomic studies. The aim of the present study was to investigate the pattern and functional characterization of CNVs in five characteristic chicken breeds, which will be important for future studies associating phenotype with chicken genome architecture.

**Results:**

Using a commercial 385 K array-based comparative genomic hybridization (aCGH) genome array, we performed CNV discovery using 10 chicken samples from four local Chinese breeds and the French breed Houdan chicken. The female Anka broiler was used as a reference. A total of 281 copy number variation regions (CNVR) were identified, covering 12.8 Mb of polymorphic sequences or 1.07% of the entire chicken genome. The functional annotation of CNVRs indicated that these regions completely or partially overlapped with 231 genes and 1032 quantitative traits loci, suggesting these CNVs have important functions and might be promising resources for exploring differences among various breeds. In addition, we employed quantitative PCR (qPCR) to further validate several copy number variable genes, such as prolactin receptor, endothelin 3 (*EDN3*), suppressor of cytokine signaling 2, CD8a molecule, with important functions, and the results suggested that *EDN3* might be a molecular marker for the selection of dark skin color in poultry production. Moreover, we also identified a new CNVR (chr24: 3484617–3512275), encoding the sortilin-related receptor gene, with copy number changes in only black-bone chicken.

**Conclusions:**

Here, we report a genome-wide analysis of the CNVs in five chicken breeds using aCGH. The association between *EDN3* and melanoblast proliferation was further confirmed using qPCR. These results provide additional information for understanding genomic variation and related phenotypic characteristics.

**Electronic supplementary material:**

The online version of this article (doi:10.1186/1471-2164-15-934) contains supplementary material, which is available to authorized users.

## Background

Genetic variation occurs in many different ways, ranging from large microscopically visible chromosome anomalies to single nucleotide changes. The subset of potential genetic variations, deletions, insertions, duplications, and complex multi-site variants, is collectively referred to as copy number variants (CNVs) [1]. CNVs range from approximately 50 bp to several Mb in size [2–4] and might exhibit potentially larger effects through the disruption of genes and alteration of gene dosages, disruption of coding sequences and perturbation of long-range gene regulation [5]. In the last decade, many studies have been conducted on the distribution, function, and role of CNVs in diseases involving DNA segments in the human genome [2, 3, 5–15]. Recently, genome-wide CNVs have not only been identified in humans but also in domestic animals, such as cattle [8, 16–19], sheep [20], goat [21], pig [22–25], and poultry [26–35].

China has a wide variety of indigenous chicken breeds, and chicken genomics is likely to have major applications and benefits in agriculture, comparative genomics, evolutionary biology, systematics, and models of development and human disease [26]. Previous studies have reported that CNVs are responsible for the phenotypic changes in chicken. Examples of phenotypes associated with CNVs in the chicken include late feathering on chromosome Z (GGAZ) [36], pea comb on GGA1 [37], dark brown plumage color on GGA1 [38], and dermal hyperpigmentation on GGA20 [39]. Furthermore, there is a large genetic distance between Chinese and European chicken populations, which facilitates the detection of fruitful breed-specific CNVs. However, compared with humans and other model organisms, there is limited research on the extent and impact of CNVs in the chicken genome. The aim of the present study was to investigate the extent and pattern of CNVs in five characteristic chicken breeds. The CNVs detected herein are complementary to the CNV map in the chicken genome, and this information will be important for future studies associating phenotype with genome architecture.

## Results

### Genome-wide detection of CNVs

**Figure 1:**
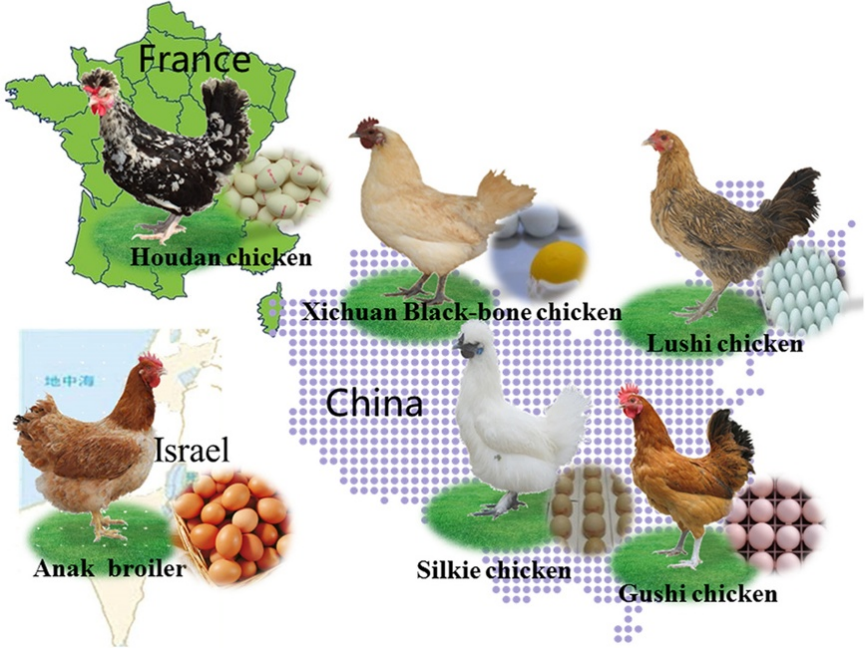
**The character of five various chicken breeds.**

Using a commercial 385 K array-based comparative genomic hybridization (aCGH) genome array (Roche NimbleGen, Inc., Madison, WI, USA), we performed CNV discovery using 10 chicken samples from four Chinese local breeds including Xichuan Black-bone chicken (XC), Silkie chicken (SK), Lushi chicken (LS), and Gushi chicken (GS), and one French breed Houdan chicken (HD) (Figure 1). Based on the WUGSC2.1/galGal3 genome sequence, a total of 1743 CNVs were identified in the genomes of these chicken breeds. After eliminating probes with uncertain chromosomal loci, we identified 446 non-redundant CNVs for the chicken autosome GGA1-28 and the Z sex chromosome. The mean and median lengths of the CNVs were 45.4 and 25.0 kb, respectively, ranging from 9.6 kb to 1.5 Mb in length. Among these segments, 171 CNVs involved a DNA sequence gain, while 275 CNVs involved a DNA sequence loss. The total number of CNVs for each breed was 91 in XC, 74 in SK, 81 in LS, 111 in GS, and 89 in HD (Additional file 1).

After aggregating the overlapping CNVs, a total of 281 CNVRs across the WUGSC2.1/galGal3 genome sequence were identified, covering 12.8 Mb of the chicken genome and 1.07% of the entire chicken genome. The mean and median sizes of the CNVRs were 45.6 and 25.0 kb, respectively. Most of the CNVRs (76.5%) were detected in a single individual, while the remaining CNVRs (33.5%) were identified in more than one individual (Additional file 1). We identified only one CNVR (chr11: 14877827–14917568) present in all the ten individuals tested. Compared with previous studies in the chicken [28, 29, 31–34], 114 (23.13%) CNVRs overlapped with previously reported CNVRs (Table 1), while 216 (76.87%) CNVRs were reported for the first time. Among the 114 overlapping CNVRs, 49 CNVRs have been identified in all studies, and 65 CNVRs have been reported in various studies (Additional file 2).

**Figure 2:**
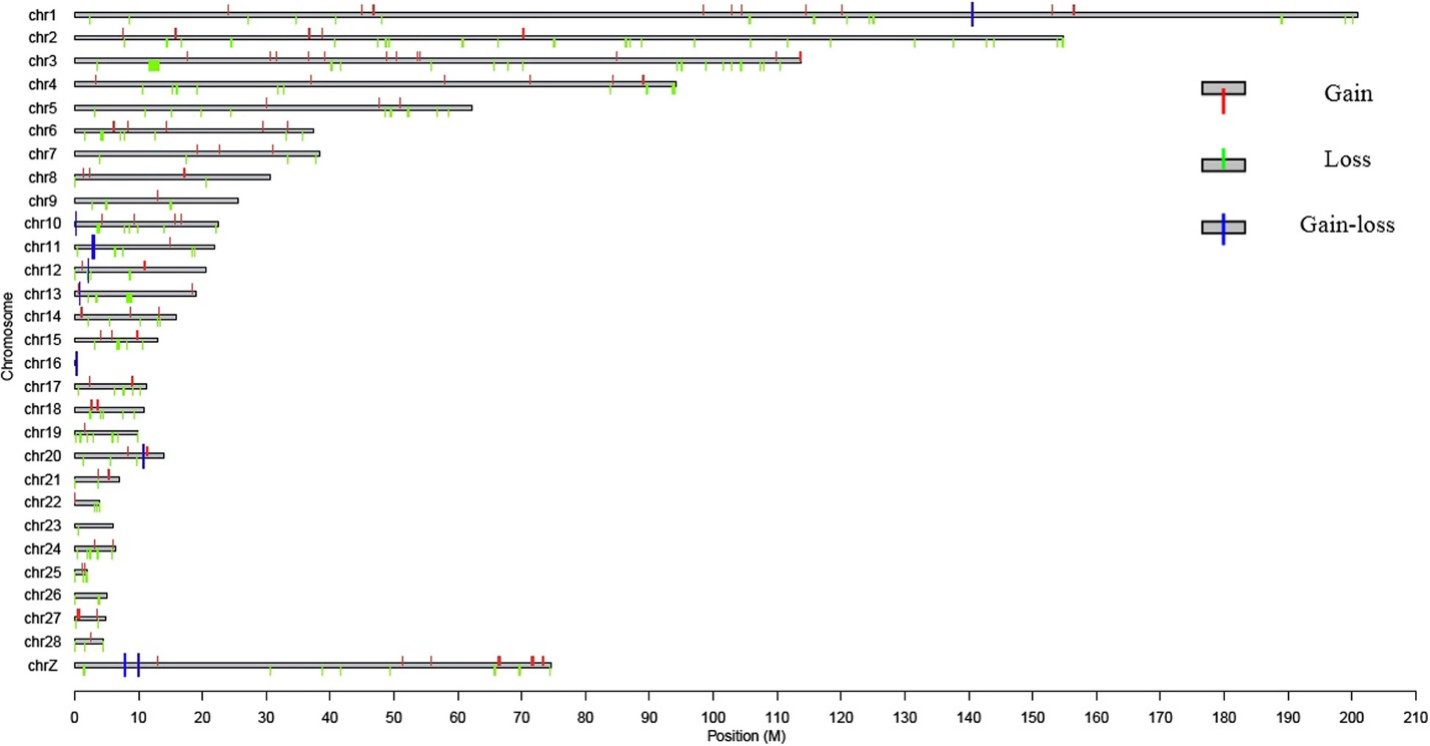
**The chromosomal locations of 281 CNVRs in local chicken breeds.** The y-axis values denote chromosome names, and the x-axis values denote chromosome positions in Mb, where the size of the color region represents the length of the CNVRs.

The 281 CNVRs identified were present on all chicken GGA1-28 autosomes and sex chromosome Z. A total of 159 (57%) CNVRs were located on chromosome 1–6, chromosome 10 and sex chromosome Z, while fewer CNVRs were located on chromosome 16 and chromosome 23. Concerning copy number status, 181 (64%) CNVRs involved a sequence loss, 91 (32%) CNVRs involved a sequence gain, and the remaining 9 (3%) CNVRs involved both a sequence loss and a sequence gain within the same region (Figure 2).

**Table 1:** **Comparison of chicken CNVRs identified in this study and in previous studies using 385 K NimbleGen whole genome-tiling arrays**

Study	Chicken CNVRs identified in this study and in previous studies								Overlaps with this study			
	Platform	Samples	Breeds/Species	CNVR	Range (kb)	Median (kb)	Mean (kb)	Total length (Mb)	Count*	Percentage	Total length (kb)	Percentage
Griffin et al. [34]	385 k NimbleGen	2	2	21	18.77–900.00	90.00	141.83	2.84	6	2.14	418.06	3.27
Völker et al. [29]	385 k NimbleGen	1	2	27	20.15–950.00	127.10	211.77	5.29	3	1.07	710.00	5.55
Wang et al. [32]	385 k NimbleGen	10	3	91	10.30–2030.06	42.59	157.17	15.72	14	4.98	814.91	6.37
Wang et al. [31]	400 k Agilent	6	3	130	6.20–649.12	14.43	25.70	3.34	12	4.27	339.79	2.65
Tian et al. [28]	400 k Agilent	22	11	308	5.82 –2025.34	14.6	35.1	10.8	29	10.32	818.53	6.39
Crooijmans et al. [33]	Agilent244K	64	15	1556	4.87-4365.80	26.0	45.2	70.1	50	17.79	2151.12	16.81
This study	385 k NimbleGen	10	5	281	9.63–1522.58	25.0	45.6	12.8	—	—	—	—

*:The number of CNVRs identified in the present study that have been previously reported.

After converting the location of the probes from galGal3 to galGal4 (2011 CGSC Gallus gallue-4.0/galGal4) according to the probe sequence, 309 non-redundant CNVs were identified in the five chicken breeds. The mean and median lengths of the CNVs were 40.7 and 22.5 kb, respectively. A total of 138 CNVs involved a DNA sequence gain, while 171 CNVs involved a DNA sequence loss. The lengths of the CNVs ranged from 1.6 to 320.5 kb. The total number of CNVs for each breed was 67 in XC, 66 in SK, 63 in LS, 72 in GS, and 41 in HD (Additional file 1). A total of 192 CNVRs were identified based on the galGal4 genome sequence. The length of these CNVRs ranged from 8.65 to 320 kb, with mean or median lengths of 36.1 and 22.5 kb, respectively. Concerning copy number status, 112 (58%) CNVRs involved a sequence loss, 75 (39%) CNVRs involved a sequence gain, and the remaining 5 (3%) CNVRs involved both variations (Additional file 1).

### Gene contents of the chicken CNV regions

Using the BioMart (http://www.biomart.org/) data management system, we retrieved the gene content of the CNVRs. A total of 231 genes (Additional file 3) completely or partially overlapped with previously identified CNVRs, including 221 protein-coding genes, 1 pseudo gene, 8 miRNA genes, and 1 rRNA gene. These genes were distributed among 167 of the 192 CNVRs identified, while the other CNVRs did not contain any annotated genes.

The Gene Ontology (GO) analyses revealed 87 GO terms and the Kyoto Encyclopedia of Genes and Genomes (KEGG) pathway displayed three pathways (Additional file 4). Among the 87 GO terms, four terms were statistically significant after Benjamini correction. The significant GO terms primarily involved cell adhesion, transcription factor activity, sequence-specific DNA binding, and transcription regulator activity (*P* <0.05). The analyses also revealed some enriched terms with marginal significance, involved in biological adhesion, pattern specification, embryonic morphogenesis, appendage development and limb development (*P* <0.1). The KEGG pathway analyses indicated that the genes in the CNVRs were enriched in three pathways, involving cell adhesion molecules, hematopoietic cell lineage, and leukocyte transendothelial migration, however, these terms were not statistically significant after Benjamini correction.

Next, we analyzed whether our CNVRs mapped to known QTLs in the chicken QTLdb [Release 20 (Apr 20, 2013): http://cn.animalgenome.org/cgi-bin/QTLdb/GG/index]. Because the confidence intervals for some QTLs were too large, we focused on QTLs with confidence intervals less than 10 Mb. A comparison of the overlapping CNVRs with QTLs revealed a total of 143 QTLs in 83 CNVRs, affecting a wide range of traits, including body weight, body size, carcass traits, fatness traits, Marek's disease-related traits, and reproductive traits (Additional file 5).

### Quantitative PCR analysis of selected CNV regions

**Figure 3:**
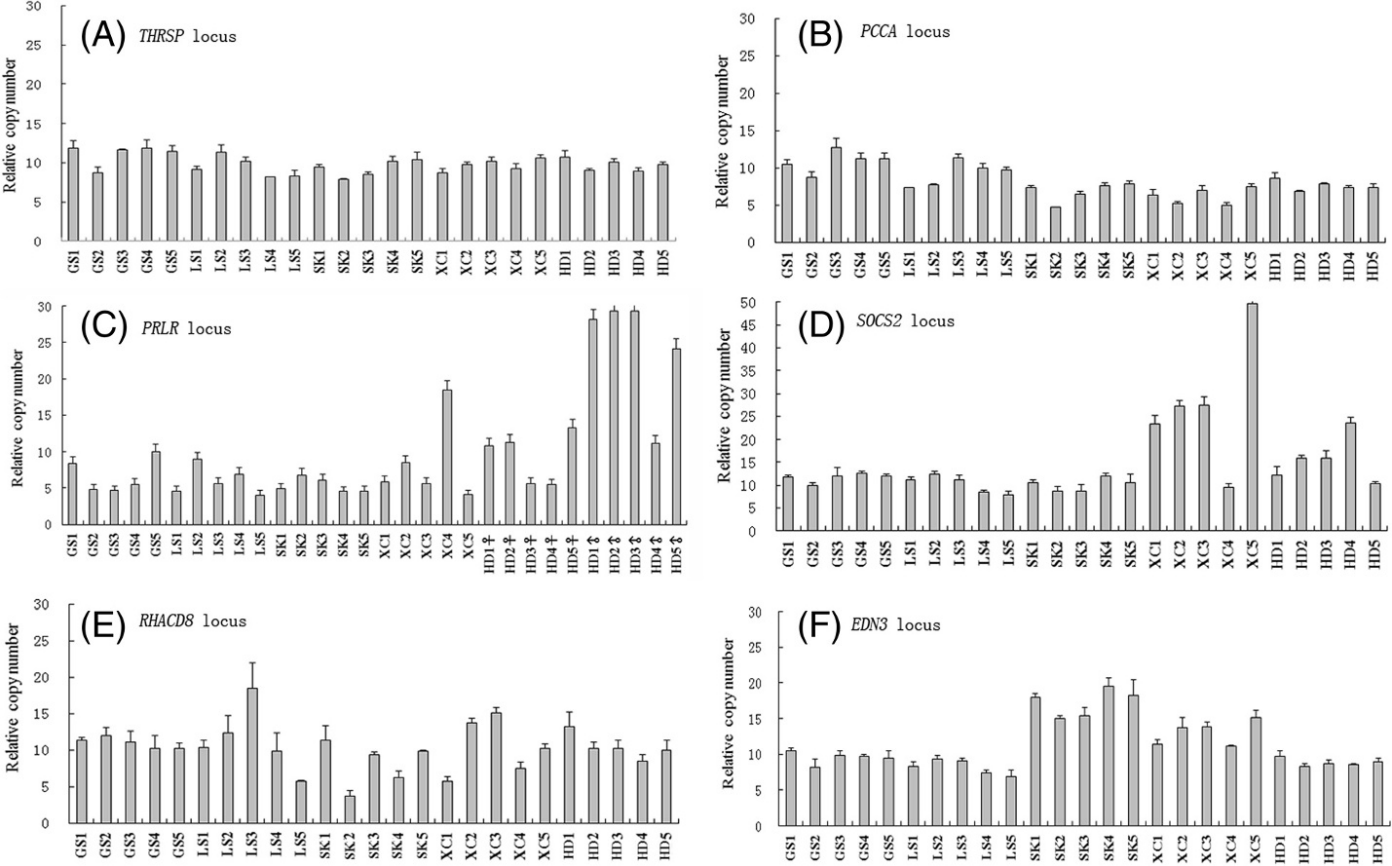
**Validation through qPCR in five test chicken breeds.** Twenty-five samples from five breeds, Gushi chicken (GS), Lushi chicken (LS), Silkie chicken (SK), Xichuan Black-bone chicken (XC) and Houdan chicken (HD), were analyzed in qPCR for the six loci. The six loci are *THRSP* locus **(A)**, *PCCA* locus **(B)**, *PRLR* locus **(C)**, *SOCS2* locus **(D)**, *RHACD8* locus **(E)** and *EDN3* locus **(F)**, respectively. Each DNA sample was diluted to 10 ng/μL, and the concentrations were verified using a spectrophotometer. Quantitative PCR analyses were processed using a standard curve method as previously described [32].

Quantitative PCR (qPCR) was performed to validate the aCGH data at six CNVR loci (Figure 3). Two (*THRSP* and *PCCA*) of the six loci served as references for no variation in copy number, while four loci (chr1: 44748534–44773370, chr4: 85228947–85423958, chr20: 11111788–11248088, and chrZ: 10472242–10661362) were CNVRs detected using an aCGH genome array in five chicken breeds. The qPCR results indicated that all four CNV loci had greater variance than the references (Figure 3), suggesting that the four loci were truly CNVs.

The qPCR results for the *THRSP* locus showed minimal variations among 25 birds (Figure 3A). We attributed these variations to random errors, including DNA dilution errors. Similar qPCR results were obtained for the *PCCA* locus in 25 birds, which also showed minimal variations among birds (Figure 3B).

In the present study, the aCGH analysis revealed the *PRLR* locus in 2 XC, 2 HD, 1 LS, and 1 GS. Subsequent qPCR analyses revealed a single copy in SK, while GS, LS, and XC had variable copy numbers, ranging from 1 to 4 copies. The results showed that female birds had 1 to 3 copies and males had 2 to 6 copies in HD (Figure 3C).

Moreover, the aCGH assay identified the *SOCS2* locus in three birds, involving a gain of copy variation. The qPCR analysis showed that the variation in copy number was far more frequent (Figure 3D) than that identified in the aCGH. GS, LS, and SK had a single copy, while HD and XC had variable copy numbers, ranging from 1 to 5 copies.

The aCGH assay revealed RHACD8 locus in 2 LS, 2 GS, 1 GF and 1 SK. The qPCR data indicated that the relative copy number for the *RHACD8* locus was highly variable among chickens. The birds with the highest copy numbers had five times as many copies as those with the lowest copy numbers. The results of the aCGH array and qPCR analyses suggested that this CNVR locus was a common variation in the autosomal region, without breed specificity (Figure 3E).

**Figure 4:**
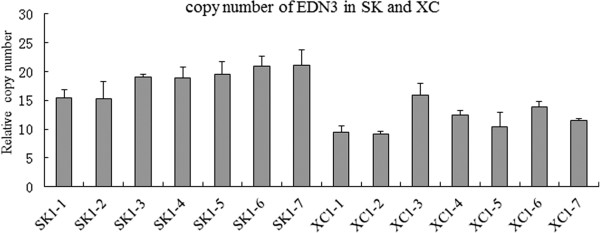
**Validation through qPCR for the** ***EDN3*** **loci in Silkie and Xichuan Black-bone chickens.** Fourteen samples from two breeds, Silkie chicken (SK) and Xichuan Black-bone chicken (XC), were analyzed using qPCR for the *EDN3* locus. Each DNA sample was diluted to 10 ng/μL, and the concentrations were verified using a spectrophotometer. qPCR analyses were processed using the same standard curve method as applied in the validation test.

**Figure 5:**
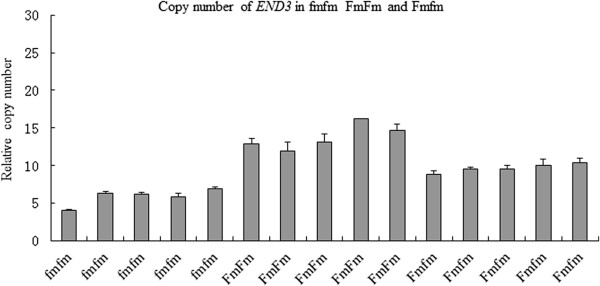
**Quantitative PCR analysis confirmed the duplication of the** ***EDN3*** **loci in three different genotypes.** Fifteen samples for the three genotypes (FmFm, fmfm, and Fmfm) were analyzed using qPCR for the *EDN3* locus. Each DNA sample was diluted to 10 ng/μL, and the concentrations were verified using a spectrophotometer. The results of the qPCR analyses were processed using the same standard curve method as performed in the validation test.

A specific duplication of a CNVR occurred on chromosome 20at base pair positions 11111788–11248088 and 11654170–11820202 encoding six annotated functional genes including the EDN3 gene. The aCGH assay revealed two loci in 2 SK and 2 XC. The qPCR results showed that GS, LS, and HD had a single copy number, and the average copy number for SK and XC was approximately 2 and 1.5-fold, respectively, higher than that of these three breeds (Figure 3 F). To confirm this result, we examined the copy numbers of 14 chickens (7 SK and 7 XC) (Figure 4). Similar qPCR results were obtained for the *EDN3* locus. To determine the copy numbers in heterozygotes (Fmfm), the genomic copy number was estimated using qPCR for three genotypes. The qPCR analysis confirmed the duplication of the *EDN3* loci in the FmFm genotype with an estimated copy number of approximately 2-fold that of wild-type individuals (fmfm genotype), while the heterozygous genotype (Fmfm) likely involved a 1.5-fold duplication (Figure 5).

## Discussion

Local Chinese chicken breeds are considered important genetic resources because of their high product quality, favorable flavor, and strong disease resistance. Here, using a tiling oligonucleotide aCGH approach, we reported the CNV survey among five unrelated characteristic chicken breeds. The number of non-redundant CNVs and CNVRs in the WUGSC2.1/galGal3 genome sequence was 446 and 281, respectively, and 309 and 192, respectively, in galGal4. Both the numbers of non-redundant CNVs and CNVRs were less in galGal4 than in galGal3 because some galGal3 probe sequences could not be successfully converted to galGal4 probe sequences. The number of CNVs and CNVRs reported were much fewer than those identified in mammals and humans.

A comparison of the CNVRs in the five breeds indicated fewer CNVs and CNVRs in HD than in the other four chicken breeds in China, and CNVR-gains were more frequent than CNVR-losses (43 losses, 46 gains) in HD. This observation might reflect the fact that the HD is different from the other four local Chinese chicken breeds in origin, appearance, and production performance.

The estimated cumulative CNVR length of 12.8 Mb (1.1% of the genome) was relatively long compared with that reported in recent studies [23, 24, 27, 34], but lower expected when considering the sample size. Indeed, Crooijmans et al. [33] reported that CNVRs represented almost 5.4% of the chicken genome when samples from 64 animals were used for testing. This difference might reflect the limited CNV coverage of the platform used in the present study, resulting in a significant underestimation of real CNVs in chickens because a limited number of individuals were surveyed. Thus, a greater number of birds should be examined to obtain a comprehensive picture of chicken CNVs. Furthermore, the incompleteness of the chicken genome assembly, suggests that a significant portion of the genome was not surveyed. The entire W chromosome was excluded from the analysis, and all probes assigned to ChrUn and other random chromosomes were also excluded.

To retrieve the information and annotation for the CNVRs based on the newest chicken genome sequence, distinct from previous studies on the gene contents of chicken CNVRs, we converted the location of the probes from galGal3 to galGal4 (2011 CGSC Gallus gallue-4.0/galGal4) according to the probe sequence, and the gene contents were processed using the galGal4 genome. The gene content analysis detected 231 Ensembl genes among the 192 identified CNVRs. Among these, 167 protein-coding genes, such as *PRLR* and *MTAP* of chrZ, *RHACD8* of chr4, *SLMO2*, *TUBB1*, and *EDN3* of chr20, etc., were annotated and reported in previous studies [32, 33]. Notably, two CNVRs (chr20: 11111788–11248088 and chr20: 11654170–11820202) were identified on chromosome 20. The distance between the loci was 419.6 kb. The CNVR chr20: 11111788–11248088 has been associated with dermal hyperpigmentation in chickens [39]. In the present study, six annotated functional genes encoding *EDN3*, zinc finger protein 831 (*ZNF831*), slowmo homolog 2 (*SLMO2*), tubulin beta-1 (*TUBB1*), cathepsin Z (*CTSZ*), partial TH1-like (Drosophila) (*TH1L*), and one miRNA were identified in this CNVR. Consistently, an association between *EDN3* and melanoblast proliferation has been previously reported in chickens [39–41].

In addition, we also identified a new CNVR involving a sequence loss of 27.7 kb in the SK (chr24: 3484617–3512275). This locus contains the gene encoding sortilin-related receptor (SORL1) [ENSGALG00000006598]. The SORL1 protein belongs to a super family of low-density lipoprotein receptors that bind apolipoprotein E and have been implicated in cholesterol metabolism [42]. In a previous study, we showed that the contents of cholesterol in Silkie eggs was higher than that in Lohmann and Lushi eggs [43], suggesting a correlation between the cholesterol content and the loss of 27.7 kb of the SORL1 gene. Therefore, this CNVR is worth further investigation.

The data obtained from GO term and pathway analyses and an examination of overlapping QTLs in the chicken QTLdb suggested that these genes function in numerous molecular functions, indicating the potential of these genes as promising resources for exploring the genetic basis of phenotypic variation within and among breeds. Specifically, 83 CNVRs partially or completely overlap with 143 QTLs, and these QTLs are involved in many important traits in chickens, including growth traits, carcass traits, meat quality traits, reproductive traits, and disease-related traits. Thus, the CNVRs identified herein might represent valuable resources for studying Chinese chicken genome diversity, including the structural variation mechanisms associated with chicken traits. Together with SNPs, CNVs will be an important complement to molecular marker and genome-wide association studies, and these variations might account for some of the missing heritability of complex traits [44].

The presence of two copies of the *PRLR* locus has been associated with the slow feathering phenotype, and the lack of one copy has been associated with the early feathering phenotype [36]. Subsequent qPCR analyses of 20 female birds showed that a single copy of this gene in SK, 1 to 2 copies in GS and LS, and 1 to 4 copies in XC. The female SK exhibited the slow feathering phenotype and the female XC, LS and GS showed both slow and fast feathering phenotypes. Moreover, in HD, the results showed that female birds have 1 to 3 copies and male birds have 2 to 6 copies. We propose that the differences in the copy numbers might reflect a gain of 2 *PRLR* loci in HD. Thus, we provide the first report that a gain of 2 *PRLR* loci also induced late feathering in HD. However, further studies are needed to examine the reliability of this finding. The *PRLP* locus is located on the Z chromosome, and because homozygote (ZZ) individuals are male and heterozygote (ZW) individuals are female, these results showed that the copy number of male birds is twice that of female birds. The results of a separate qPCR assay in HD were consistent with this idea. A sex-linked late feathering allele K containing 2 copies of the *PRLR* locus has been introduced into commercial flocks and used widely for sexing hatchlings. This K allele is incompletely dominant to the early feathering k^+^ allele containing one copy of the *PRLR* locus. Suppliers typically cross k^+^k^+^ males with KW females to generate female progeny with the early feathering phenotype k^+^W containing one copy of *PRLR*, while the male progeny have a late feathering phenotype Kk^+^ containing three copies of *PRLR*.

We observed that SK and XC were the only two breeds to show CNVs at the *EDN3* locus. Dorshorst et al. [40] reported that Fm results from an inverted duplication of two genomic regions, each greater than 100 kb, located on chromosome 20, resulting in the increased expression of the *EDN3* gene. We concluded that the dermal hyperpigmentation of these two Chinese local chicken breeds also resulted from a CNV in this region. SK and XC are closely distributed in southeast China, likely reflecting the fact that these two breeds originated from the same place or have traits that were purposely bred into different strains. To explain the observation that the copy number in SK was higher than that in XC, we propose that both heterozygotes (Fmfm) and homozygotes (fmfm) are present in XC. Using qPCR analysis, Dorshorst et al. [40] confirmed the duplication of both genomic regions in Fm birds, with an estimated copy number of approximately 1.5-2-fold that of wild-type individuals, suggesting that some Fm birds were likely heterozygous for a mutant allele comprising a 2-fold duplication. This result suggests that this locus might be a molecular marker for the selection of dark skin color in poultry production.

## Conclusions

Here, we reported a genome-wide analysis of CNVs in five chicken breeds using an aCGH array. The mapping of CNVs will contribute to association studies of economic performance. Among the four CNVRs selected for validation through qPCR, all four variations exhibited the expected copy number differences. The association between *EDN3* and melanoblast proliferation was further confirmed in the present study. Additional, in the present study, we report a new CNVR (chr24: 3484617–3512275), encoding SORL1 gene, with a copy number change only in black-bone chickens. These results provide a preliminary foundation for investigating the association between various phenotypes and CNVs. Future studies are required to assess the functional significance of CNVs and determine the impact of these variations on economic traits in chicken.

## Methods

### Ethics statements

The entire procedure for the collection of blood samples of the all animals was performed in strict accordance with the protocol approved by the Institutional Animal Care and Use Committee (IACUC) of Henan Agricultural University.

### Sample preparation

In the present study, 10 individuals were selected from five chicken breeds (two females from each breed) and used as test samples, while one female Anka broiler was used as a reference. The five chicken breeds included four Chinese indigenous breeds, Xichuan Black-bone chicken (XC), Silkie chicken (SK), Lushi chicken (LS), Gushi chicken (GS) and one French commercial breed, Houdan chicken (HD) (Figure 1).

All blood samples used in the present study were collected in 0.5 M EDTA and stored at −20°C until further DNA isolation. The DNA was isolated using the DNeasy Blood and Tissue Kit (Qiagen, Hilden, Germany). The concentration and quality of the DNA samples were quantified using a NanoDrop (NanoDrop Technologies, Wilmington, DE, USA) and 1% agarose gel electrophoresis.

Ten samples (two from each bird) were analyzed using an aCGH array and fifty-nine samples were analyzed through qPCR. Twenty-five samples (five from each breed) were analyzed for all the six loci. In addition, another five male HD were analyzed for the *PRLR* locus, and twenty-nine samples (7 SK and 7 XC; 5 SK, 5 Hisex Brown A line and 5 first filial generations of them) were analyzed for the *EDN3* locus.

### Oligonucleotide array CGH

NimbleGen chicken whole genome tiling array (Catalog Number/Design Name B3791001-00-01, galGal3_WG_CGH – Roche NimbleGen, Milton Keynes, UK) was used for all microarray experiments. This array contains 385,000 oligonucleotide probes (http://www.nimblegen.com) with a median probe spacing of 2,586 bp. Genomic DNA labeling, hybridization and array scanning were performed according to the manufacturer’s instructions. Each test DNA sample (labeled with Cy3) was cohybridized with the reference female broiler sample (labeled with Cy5). The initial data analysis (normalization and segmentation) was performed using DEVA 1.2 software (Roche-NimbleGen). Next, each WUGSC2.1/galGal3 probe sequence was converted to galGal4 genome sequences, followed by analysis using NimbleScan 2.4 software (segMNT algorithm). The technical specifics for NimbleGen software are provided at http://www.nimblegen.com/products/lit/lit.html.

### Identification of CNVs and CNVR

The high confidence calls were obtained according to Wang et al. [32], namely segments of five or more probes with mean log_2_ ratio shift from baseline greater than +/− 0.3 were flagged as candidate CNVs. Probes from uncertain chromosomal loci (Chr#-random, ChrUn-random, chrE22C19W28_E50C23, chrE64, and W chromosome in the UCSC database) were removed from the results. The CNVRs were determined after aggregating the overlapping CNVs identified across all samples according to Redon et al. [2]. We used the standards (overlap ≥1 bp) to evaluate overlaps between the results obtained in the present study and those of previous reports. In the present study, the range of overlap is 3.7-332.7 kb. Raw aCGH data for this study have been deposited in GenBank GEO database under accession number: GSE49889, http://www.ncbi.nlm.nih.gov/geo/query/acc.cgi?acc=GSE49889.

As an outdated version of this software was used in the present study (source - UCSC, build - galGal3), we first converted the galGal3 probes to galGal4 probes and subsequently performed the CNV analysis using a process similar to that described above. The functional annotation and analysis of the CNVR were based on the converted CNVRs.

### Functional annotation and analysis of the CNVR

To provide insight into the functional enrichment of the CNVs, a functional annotation was performed using the DAVID Bioinformatics Resources software, version 6.7 (http://david.abcc.ncifcrf.gov/summary.jsp) to obtain GO terms [45] and KEGG [46] pathway analyses. Because only a limited number of genes in the chicken genome have been annotated, we converted the chicken Ensembl gene IDs to orthologous human Ensembl gene IDs using BioMart software, and the statistical significance was using the modified Fisher's exact test and Benjamini correction for multiple testing. We also performed an overlap analyses between the CNVRs identified and the reported QTL regions annotated in the chicken QTL database (April 20, 2013, (http://www.animalgenome.org/cgi-bin/QTLdb/GG/index).

### CNV validation by quantitative PCR

Quantitative PCR primers for *EDN3* were designed using Primer 5.0 software. The primers for *SOCS2*, *THRSP*, *PCCA*, *RHACD8* and *PRLR* were designed as previously described [31, 32]. The primer sequences are listed in Additional file 6.

The qPCR assays conducted using SYBR SELECT MASTER MIX (Life Technologies, California, USA). The reactions were performed in 20 μl containing 20 ng of genomic DNA and 0.5 μM of each primer. The cycling conditions included pre-incubation at 50°C for 2 min and 95°C for 2 min, followed by 40 cycles of amplification (95°C for 30 s, 57°C for 30 s and 72°C for 30 s). The primers were validated using melting curve, amplification and standard curve analyses and no-template control reactions. For the standard curve analysis, one DNA sample was serial diluted to 2.5, 5, 10, 20, 40 and 80 ng/μl and measured again using a spectrophotometer. Each concentration was analyzed in three-fold through qPCR to determine the amplification efficiency.

Each genomic DNA sample was diluted to 10 ng/μl in double-distilled water and assessed using qPCR in triplicate reactions. The qPCR was performed using the iCycler system (Bio-Rad, Hercules, CA), with individual PCR tubes (Bio-Rad, cat# TLS0801, Hercules, CA). In the qPCR assay, the relative copy numbers were assigned after comparing the Ct values with the standard curve and the amount of copies in 1 ng of reference DNA (assumed as one unit).

## Electronic supplementary material

Additional file 1: **Comparison of CNVs and CNVRs between galGal3 and galGal4 genome sequences.** (DOC 39 KB)

Additional file 2: **Comparison of the CNVRs identified in the present study with those reported in other studies.** (XLSX 37 KB)

Additional file 3: **Information on the genes in the CNVRs identified in the present study.** (XLSX 31 KB)

Additional file 4: **Gene ontology and pathway analyses of genes in the CNVRs identified in the present study.** (XLSX 21 KB)

Additional file 5: **Previously reported QTLs that overlapped with the CNVRs identified in the present study.** (XLSX 149 KB)

Additional file 6: **Quantitative PCR primers for the six CNVR loci examined in the present study.** (XLSX 11 KB)

## Abbreviations

aCGH: Array-based comparative genome hybridization
CNV: Copy number variation
CNVR: CNV region
Mb: Megabases
PCR: Polymerase chain reaction
qPCR: Quantitative PCR
SNP: Single nucleotide polymorphism
QTLs: Quantitative trait loci
PCCA: Propionyl coenzyme A carboxylase
PRLR: Prolactin receptor
SORL1: Sortilin-related receptor
THRSP: Thyroid hormone responsive
SOCS2: Suppressor of cytokine signaling 2
RHACD8: CD8a molecule
XC: Xichuan Black-bone chicken
SK: Silkie chicken
LS: Lushi chicken
GS: Gushi chicken
HD: Houdan chicken
GO: Gene ontology
KEGG: Kyoto encyclopedia of genes and genomes.
