# Supplementary material for: Identification and functional characterization of copy number variations in diverse chicken breeds
Source: BMC Genomics. 2014 Oct 25;15(1):934. doi: 10.1186/1471-2164-15-934 (PMC4226851; doi:10.1186/1471-2164-15-934)
Supplement: Supplementary file 1 — Additional file 1: Comparison of CNVs and CNVRs between galGal3 and galGal4 genome sequences. (DOC 39 KB) [file 12864_2014_6636_MOESM1_ESM.doc]

**Additional file 1. Comparison of CNVs and CNVRs between galGal3 and galGal4 genome sequence**

| Item | CNV | | CNVR | |
| --- | --- | --- | --- | --- |
|  | galGal3 | galGal4 | galGal3 | galGal4 |
| The number of non redundant CNVs | 446 | 309 | 281 | 192 |
| mean lengths(kb) | 45.4 | 40.7 | 45.6 | 36.1 |
| median lengths(kb) | 25.0 | 22.5 | 25.0 | 22.5 |
| The maximum length (kb) | 1522 | 320.5 | 1522 | 320.5 |
| The minimum length (kb) | 9.6 | 1.6 | 9.6 | 8.7 |
| The number of Gain | 171 | 138 | 91 | 75 |
| The number of Loss | 275 | 171 | 181 | 112 |
| The number of Loss and Gain |  |  | 9 | 5 |
| The number in one bird |  |  | 215 | 141 |
| The number at least in two birds |  |  | 66 | 51 |
| The CNVs of XC | 91 | 67 | 86 | 66 |
| The CNVs of WJ | 74 | 66 | 72 | 64 |
| The CNVs of LS | 81 | 63 | 79 | 64 |
| The CNVs of GS | 111 | 72 | 105 | 59 |
| The CNVs of GF | 89 | 41 | 56 | 53 |
| Total CNV lengh (Mb) | 20 | 12.6 | 12.8 | 6.94 |
